# Emerging Role of Angiotensin Type 2 Receptor (AT2R)/Akt/NO Pathway in Vascular Smooth Muscle Cell in the Hyperthyroidism

**DOI:** 10.1371/journal.pone.0061982

**Published:** 2013-04-24

**Authors:** Maria Alícia Carrillo-Sepúlveda, Graziela S. Ceravolo, Cristina R. Furstenau, Priscilla de Souza Monteiro, Zuleica Bruno-Fortes, Maria Helena Carvalho, Francisco R. Laurindo, Rita C. Tostes, R. Clinton Webb, Maria Luiza M. Barreto-Chaves

**Affiliations:** 1 Laboratory of Cell Biology and Functional Anatomy, Department of Anatomy, Institute of Biomedical Sciences, University of Sao Paulo, Sao Paulo, Brazil; 2 Laboratory of Hypertension, Institute of Biomedical Sciences, University of Sao Paulo, Sao Paulo, Brazil; 3 Vascular Biology Laboratory of Heart Institute, University of Sao Paulo, Sao Paulo, Brazil; 4 Department of Physiology, Georgia Health Science University, Augusta, Georgia, United States of America; University of Bonn, Germany

## Abstract

Hyperthyroidism is characterized by increased vascular relaxation and decreased vascular contraction and is associated with augmented levels of triiodothyronine (T3) that contribute to the diminished systemic vascular resistance found in this condition. T3 leads to augmented NO production via PI3K/Akt signaling pathway, which in turn causes vascular smooth muscle cell (VSMC) relaxation; however, the underlying mechanisms involved remain largely unknown. Evidence from human and animal studies demonstrates that the renin-angiotensin system (RAS) plays a crucial role in vascular function and also mediates some of cardiovascular effects found during hyperthyroidism. Thus, in this study, we hypothesized that type 2 angiotensin II receptor (AT2R), a key component of RAS vasodilatory actions, mediates T3 induced-decreased vascular contraction. Marked induction of AT2R expression was observed in aortas from T3-induced hyperthyroid rats (Hyper). These vessels showed decreased protein levels of the contractile apparatus: α-actin, calponin and phosphorylated myosin light chain (p-MLC). Vascular reactivity studies showed that denuded aortic rings from Hyper rats exhibited decreased maximal contractile response to angiotensin II (AngII), which was attenuated in aortic rings pre-incubated with an AT2R blocker. Further study showed that cultured VSMC stimulated with T3 (0.1 µmol/L) for 24 hours had increased AT2R gene and protein expression. Augmented NO levels and decreased p-MLC levels were found in VSMC stimulated with T3, both of which were reversed by a PI3K/Akt inhibitor and AT2R blocker. These findings indicate for the first time that the AT2R/Akt/NO pathway contributes to decreased contractile responses in rat aorta, promoted by T3, and this mechanism is independent from the endothelium.

## Introduction

Thyroid diseases, which are estimated to affect 10% of the population [1], [2] are accompanied by profound cardiovascular changes. In particular, hyperthyroidism induces a high-output state, with a marked decrease (up to a 50 percent) in systemic vascular resistance (SVR), possibly due to local release of vasodilators in peripheral tissues as a consequence of the elevated tissue metabolism [3]. The diminished SVR seen in hyperthyroidism has been attributed to a direct effect of T3, the biologically active form of thyroid hormone, on vascular endothelium [4]. However, the underlying mechanisms associated with the T3 actions on the vascular smooth muscle cell (VSMC) are still unclear. Our group has demonstrated that T3 promoted marked NO production in VSMC by rapid nongenomic actions, which in turn contributed to vascular relaxation [5], suggesting the VSMC as a specific target of T3, which may represent an important factor for local control of vascular function.

Several lines of evidence suggest an important contribution of the renin-angiotensin system (RAS) to the actions of the thyroid hormones (TH) [6]–[9]. Two isoforms for angiotensin II receptor have been identified in VSMC: type 1 receptor (AT1R) and type 2 receptor (AT2R) [10], [11]. AT2R is upregulated in certain pathological conditions such as hypertension, vascular injury, inflammation [12] and also in hyperthyroidism [8]. The AT2R mediates cellular differentiation and growth, and in some circumstances opposes the actions of AT1R stimulation [13]–[15] and therefore, it is important in reducing tissue remodeling and disease progression. In addition, AT2R promotes relaxation in rat isolated resistance arteries and aortas [16], [17] via activation of a vasodilatory cascade involving bradykinin (BK), nitric oxide (NO), and guanosine cyclic 3′,5′-monophosphate (cGMP), counteracting AT1R-induced contraction [18], [19].

Based on evidence that AT2R stimulates NO production [20] and that NO production is increased in VSMC by nongenomic action of T3 [5], as well as data showing that thyroid hormone induces an upregulation of AT2 receptor in cardiac myocytes [8], we hypothesized that AT2R contributes to the decreased VSMC contraction observed during hyperthyroidism condition, which may represent an important mechanism to vascular relaxation observed in this pathology. In this study we evidenced for the first time that AT2R/Akt/NO pathway represents a novel mechanism associated with the decreased VSMC contraction promoted by thyroid hormone.

## Methods

### Ethics Statement

All procedures were performed in accordance with the Guiding Principles in the Care and Use of Animals, approved by Ethics Committee for Animal Research of the Institute of Biomedical Sciences, University of Sao Paulo- Brazil and also by Georgia Health Sciences University Committee on the Use of Animals in Research and Education published by the US National Institutes of Health.

### Animals and Experimental Procedures

Adult, male Wistar rats weighing 200–260 g were housed under a temperature-and light-controlled environment (22±1°C on a 12 h light/dark cycle at 60% humidity) and were given free access to standard rat chow diet and water *ad libitum.* Rats were randomized into two groups: control and hyperthyroid (Hyper). Rats were induced to hyperthyroidism by daily injections of T3 (7 µg/100 g BW in 0.01 mM NaOH, i.p.) for 14 days, while control rats received a daily injection of vehicle. Unless otherwise stated, the rats were killed by decapitation 24 h after the last dose of T3 (Sigma, St Louis, MO). Blood samples were collected in order to evaluate the serum levels of thyroid hormone (TH) and confirm the hyperthyroid status of the animals. The ratio between heart weight (HW) and tibial length (TL) (in mg/mm) was used as an index of cardiac hypertrophy, usually observed in hyperthyroid condition.

Thoracic aortas were isolated and used for functional studies and molecular assays.

### Hemodynamic Parameters

To fully characterize our animal model of hyperthyroidism, body weight (BW) was evaluated daily and indirect systolic blood pressure (SBP) and heart rate (HR) were determined at the same time of day by tail-cuff plethysmography (Kent Scientific, Litchfield, CT). Rats were familiarized with the apparatus for a total of 7 days before the measurements were taken. The final determinations of SBP and BW were made immediately before the animals were sacrificed.

### Serum TH Measurements

Trunk blood was collected without anticoagulant, centrifuged at 3,000 rpm for 15 min at 4°C, and stored at −20°C. Levels of free T3 and thyroxine were determined in the serum of control and hyper group using a commercial radioimmunoassay kit (CIS Bio International, Gif-sur-Yvette, France).

### Vascular Functional Studies

In one set of experiments, after euthanasia with isoflurane (via nasal 5% in 100% O_2_, thoracic aortas were removed and cleaned from fat tissue in Krebs solution (130 mmol/L NaCl, 14.9 mmol/L NaHCO3, 4.7 mmol/L KCl, 1.18 mmol/L KH2PO4, 1.17 mmol/L MgSO_4_-7H_2_O, 1.56 mmol/L CaCl_2_-2H_2_O, 0.026 mmol/L EDTA, 5.5 mmol/L glucose). Aortas were cut into rings (2 mm in length), were carefully mounted in a myograph for isometric tension recordings and were equilibrated in Krebs solution for 30 min, gassed with 5% CO_2_ in O_2_ to maintain a pH of 7.4, as described previously [5]. Aortas rings were placed under resting tension (30 mN, previously determined) for 1 hour with frequent buffer changes until equilibrated. In all experiments the endothelium was mechanically removed by gently rolling the lumen of the vessel on a thin metallic wire. The absence of the endothelium was verified using acetylcholine (ACh) in phenylephrine (PE) pre-contracting aortic ring preparations. Aortic rings producing less than 10% relaxation to Ach were regarded as indication of successful denudation. Cumulative concentration-response curves to angiotensin II (Ang II, 0.1 nM - 0.1 µM) were performed in the absence or presence of AT2R blocker, PD123319 (0.1 µM, 30 min incubation). This blocker has a high affinity for AT2R and a low affinity for the AT1R [21].

### Cell Cultures

Isolation, characterization and maintenance of cultured rat aortic VSMC have been previously described [5], [22]. Briefly, cells were maintained in DMEM containing 10% FBS and antibiotics. After reaching confluence, VSMC were made quiescent by serum-deprivation for 24 hours and then stimulated with T3 (0.1 µM) for 24 hours. In some experiments cells were pretreated with PD123319 (0.1 µM) [23], or with Wortmannin (100 nM), a selective inhibitor of PI3K/Akt [24] for 30 min.

### mRNA and Protein Expression

Total RNA from cultured VSMC was isolated with Trizol Reagent (Invitrogen, CA, USA). For reverse transcription, we employed 1 µg of total RNA using SuperScript II Reverse Transcriptase (Invitrogen). Real-time RT-PCR was performed in a thermocycler (Corbett Research, Sydney, Australia) using the SYBR Green PCR master mix (Invitrogen) according to the manufacturer’s recommendations. The following primer sequences were used for AT2R: 5′- CCT TCT TGG ATG CTC TGA CC -3′ and 5′- TGG AGC CAA GTA ATG GGA AC -3′, while those for β-actin were: 5′- TAT GCC AAC ACA GTG CTG TCT GG -3′ and 5′- TAC TCC TGC TTC CTG ATC CAC AT -3′. Samples were run in duplicate, and the real-time RT-PCR data were normalized to β-actin, since we have previously confirmed that variations in TH levels do not alter β-actin mRNA levels [8].

Immunoblottings were performed as previously described [5]. Extracted protein (30 µg) from aorta or cultured VSMC was subjected to SDS-PAGE (10%) gel electrophoresis and transferred to a nitrocellulose membrane. The membranes were incubated with primary antibodies (Santa Cruz Biotechnology, except for α-actin, from Sigma) overnight at 4°C and a mouse monoclonal α-actinin specific antibody was used as an internal control. The primary antibodies used in this study were against RAS components (AngI/AngII, AT1R, AT2R), contractile proteins (α-actin, calponin, p-MLC and Total MLC), and Akt signaling pathway (Thr^308^-Akt, Ser^473^-Akt, Total Akt). Bound proteins were detected using a chemi-luminescence reaction kit (Amersham Pharmacia Inc., Uppsala, Sweden).

### NO Detection Assay

The release of NO from VSMC stimulated with T3 (0.1 µM) for 24 hours was measured as nitrite (NO_2_^−^) accumulation in the culture media and media from untreated VSMC was used as control, as previously described [25]. The blocker of AT2R or PI3K inhibitor (PD123319, 0.1 µM or Wortmannin, 0.1 µM, respectively), were added 30 minutes before T3 stimulation. We analyzed the chemi-luminescence reaction between ozone and the NO generated by reduction of the sample with vanadium chloride in acid at 95°C using an NO analyzer (Model 208A; Sievers Instruments Inc., Boulder, CO) according to the manufacturer protocols. NO_2_^−^ levels were corrected for total protein content of VSMC extracts determined by Bradford method [26]. The rates of NO_2_^−^ accumulation were expressed as micromol per gram of protein.

In other experiments, non destructive NO was detected using the NO-specific fluorescent dye, 4,5-diaminofluorescein diacetate (DAF-2, Sigma, St. Louis, MO, USA) as previously reported [5]. VSMC were loaded with DAF-2 (10 µM) for 30 min and then treated with T3 (0.1 µM) for 24 hours. To investigate the participation of PI3K/Akt pathway in T3 induced-NO production, VSMC were pre-incubated with wortmannin (0.1 µM) for 30 min. Images were obtained by fluorescence microscope (Axioskop, Zeiss, Göttigen, Germany). Fluorescence intensity in each cell was quantified by pixel intensity of the cell using image software (KS-300 Software, Zeiss). Background subtraction was performed for each image prior to the quantification. The intensity of fluorescence is directly proportional to NO concentration present in the media [27].

### Statistical Analysis

All data are expressed as mean ± SD, unless otherwise indicated. Contractions were recorded as changes in the displacement (mN) from baseline. Concentration-response curves were fitted using a nonlinear interactive fitting program (Graph Pad Prism 4.0; GraphPad Software Inc.), and two pharmacological parameters were obtained: the maximal effect generated by the agonist (or E_max_) and –log EC_50_ (or pD_2_). Statistical analyses were performed using One-way ANOVA or Student’s test. Post-hoc comparisons were performed using Bonferroni’s test. Statistical significance was accepted at p<0.05.

## Results

### Validation of Hyperthyroidism Experimental Model in Rats

To determine whether the T3-treated rats were induced successfully to hyperthyroidism, the serum levels of free T3 and T4 were measured. Additionally, some of the typical alterations present in hyperthyroidism [28] such as cardiac hypertrophy, systolic blood pressure, heart rate, and body weight were also determined (Table 1). T3-treated rats exhibited increased T3 and decreased T4 serum levels, which confirms the hyperthyroid status. Similar to humans, hyperthyroid rats showed an augmented metabolic condition characterized by decreased body weight and also tachycardia. T3 treatment led to cardiac hypertrophy determined by an increase in the heart weight to tibia length ratio. No changes were observed in systolic blood pressure in control animals during treatment, while T3 induced a significant increase (approximately 15%) after 14 days of treatment. Taken together these results confirm the efficiency of T3 treatment inducing hyperthyroidism in these rats.

**Table 1:** Experimental groups at baseline and after 14 days of T3-treatment.

		*Co Control*		*Hy Hyperthyroid*	
*Parameter*	*0 Day*	*14 Days*	*0 Day*	*14 Days*	
Body weight (g)	271.5±9.8	368.3±16.8	268.5±14.2	272.1±12.4*	
SBP (mmHg)	121.3±6.2	126.6±7.1	123.6±6.3	139.7±7.7*	
HR (beats/min)	374±17	383±15	372±20	448±12*	
HW/TL (mg/mm)		18.8±1.3		28.6±2.1*	
Free T3 (ng/ml)		0.42±0.11		1.31±0.56*	
Free T4 (ng/ml)		31.9±3.35		8.8±0.68*	

Values are expressed as mean ± SD; n = 12 per group. BP indicates blood pressure; HW, heart weight; BW, body weight; TL, tibial length; T3, triiodothyronine; T4, thyroxine.

* p<0.05, vs. Control.

### T3-treatment Decreases Contractile Protein Levels in Aortas

**Figure 1:**
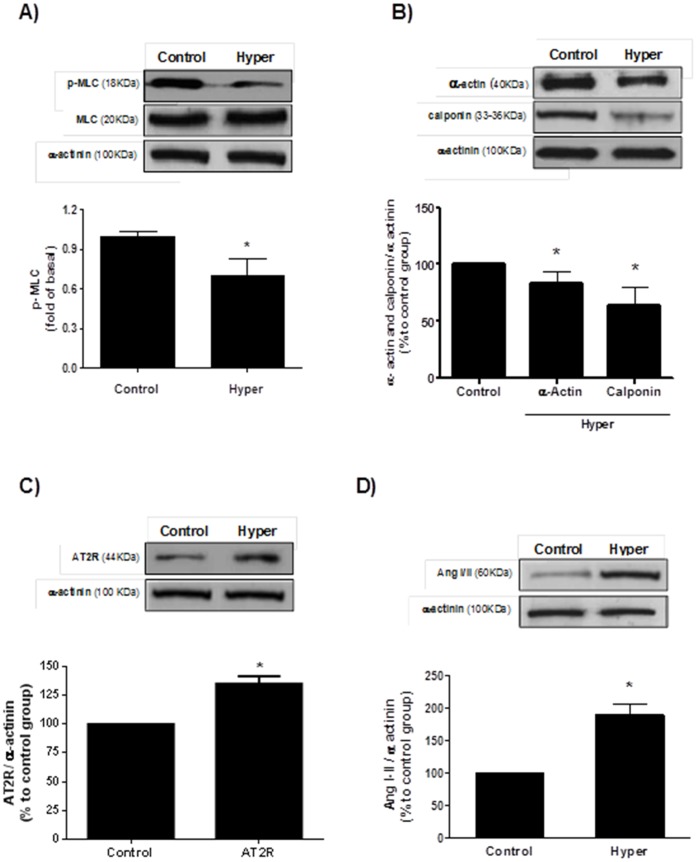
Effect of Hyperthyroidism (Hyper) in the protein expression. Hyper reduced the expression of contractile proteins: p-MLC (A), α-actin and calponin (B). In addition, Hyper was associated with increased AT2R (C) and AngI/II (D) levels in aortas. Top: representative blots. Bottom: Densitometry values. Data are expressed as mean ± SD (n = 6) per group, *p<0.05 *vs.* control.

One likely explanation for the vascular relaxation present in hyperthyroidism is a reduction in proteins related to smooth muscle contraction. Indeed, phosphorylated MLC levels (p-MLC), a well-known marker of contraction, were decreased by 30% in aortas from T3-treated rats compared to vehicle treated (Figure 1A). Moreover, decreased expression of α-actin and calponin, both essential proteins in the contractile mechanism of smooth muscle, were observed in the aortas from T3-treated rats (Figure 1B).

### T3-treatment Promotes Upregulation of AT2R

Since it has been demonstrated that AT2R play a physiological role in the hypertrophy of cardiac myocytes under hyperthyroidism conditions [8] and because AT2R is directly associated with the production of the vasodilatory cascade composed by bradykinin, nitric oxide and guanosine cyclic 3′,5′-monophosphate (cGMP) that mediates vasodilatation [18], [29] we evaluated the AngI/AngII and AT2R levels in aortas from hyperthyroid animals. Hyper group presented a significant upregulation of AT2R expression (Figure 1C) and increased local AngI/AngII levels compared to control (Figure 1D).

### T3-treatment Decreases Contraction to Ang II in the Aorta via AT2R

**Figure 2:**
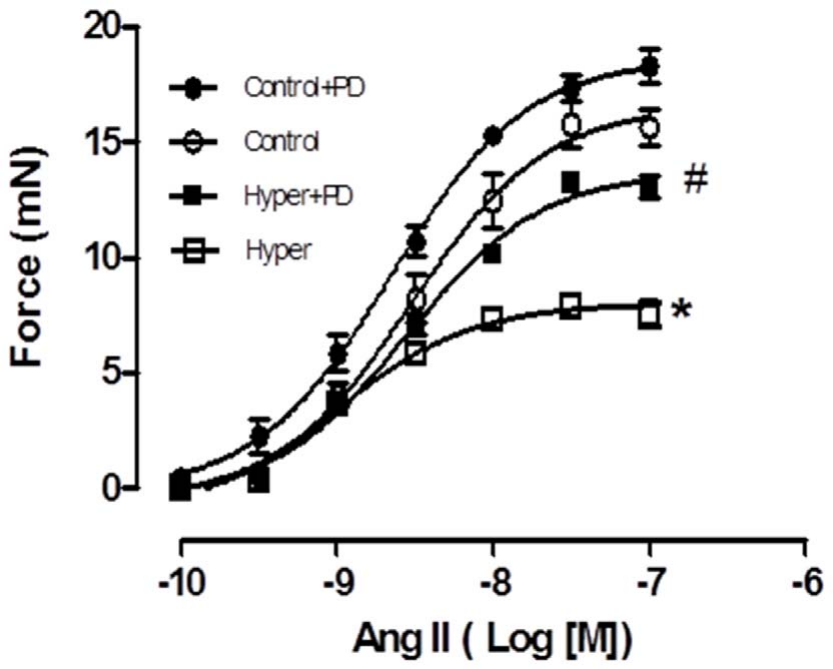
AT2R mediates endothelium-independent decreased contraction in aortas from hyperthyroid rats. Contractile response to AngII (from 0.1 nM to 0.1 µM) is decreased in hyper denuded-aortas (open square) when compared to control group (open circle). The specific blockade of AT2R with 0.1 µM PD123319 (filled square) reverses this effect when compared to control group pretreated with PD (filled circle). Data are represented as mean ± SEM (n = 4 per group, *p<0.05 *vs.* control group, #p<0.05 *vs.* control+PD).

In order to determine whether the upregulation of AT2R contributes to T3-induced relaxation, concentration-response curves to angiotensin II (Ang II) were performed in endothelium-denuded aortic rings from Hyper and control rats, in the presence or absence of PD123319, a specific AT2R blocker. Ang II produced concentration-dependent contraction in all tested aortic rings. Aortas from Hyper rats displayed decreased contraction to Ang II compared with control aortas (E_max_ 6.73±0.37 vs. 15.2±0.56 mN, p<0.05, respectively) and this effect was attenuated in the aortas pre-incubated with PD123319 (12.45±0.41 mN, p<0.05) (Figure 2). No differences were observed in the pD_2_ among the groups. These data suggest AT2R is involved in decreased contraction to AngII in hyperthyroid rats.

### T3 Increases AT2R Gene and Protein Expression Levels in Cultured VSMC

**Figure 3:**
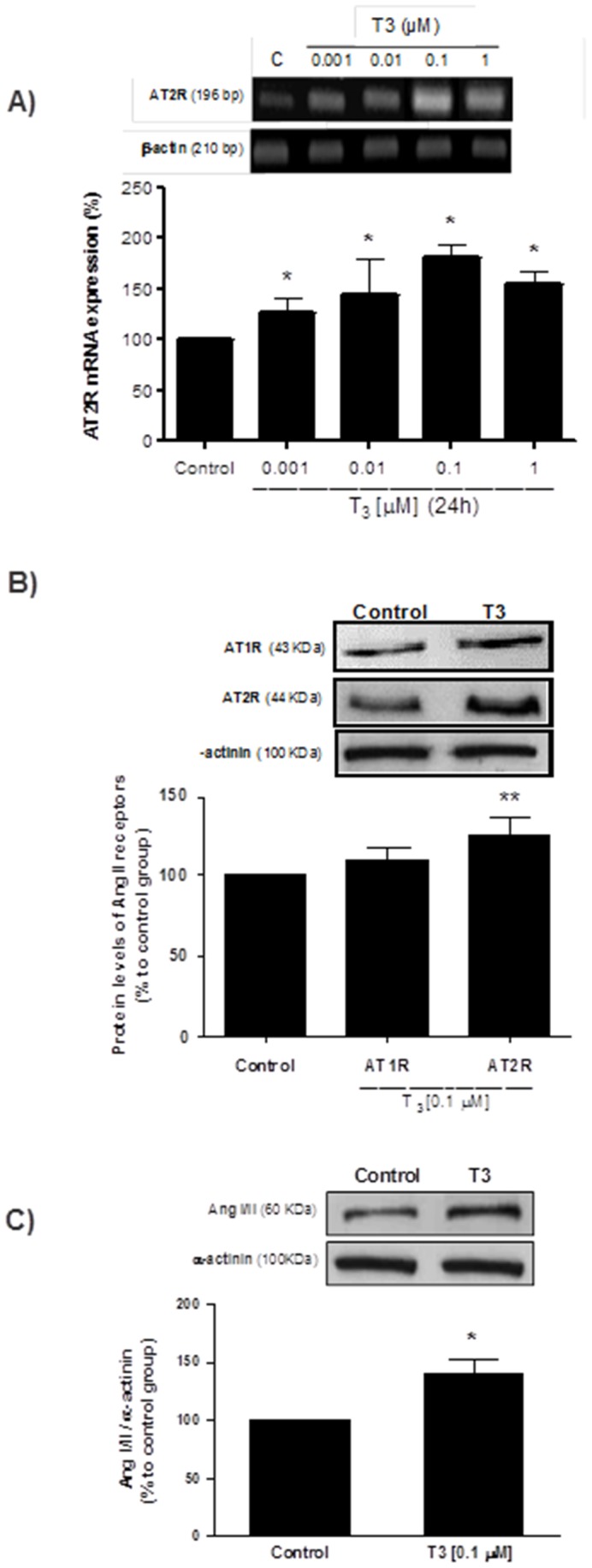
T3 upregulates AT2R in VSMC. Concentration-dependent T3-induced increased AT2mRNA in cultured VSMC (A).VSMC treated with T3 (0.1 µm) for 24 hours leaded to an increased AT2R (B) and AngI/II (C) protein expression. Data represent mean ± SD in %, considering the control situation as 100% (n = 5 per group, *p<0.05 *vs.* control).

Based on the effects of hyperthyroidism on AT2R expression in aortas, we examined the effect of T3 treatment in cultured VSMC. The AT2R mRNA levels were significantly increased in a T3 concentration-dependent manner (from 0.001 µM to 1.0 µM) (Figure 3A). Further, VSMC stimulated with T3 (0.1 µM) for 24 hours showed augmented AT2R and unchanged AT1R protein expression (Figure 3B), which was accompanied by increased AngI/II levels (Figure 3C). These results indicate that T3 plays important role in local activation of RAS.

### AT2R Mediates the T3-induced Diminution of Contractile Protein Expression in Cultured VSMC

**Figure 4:**
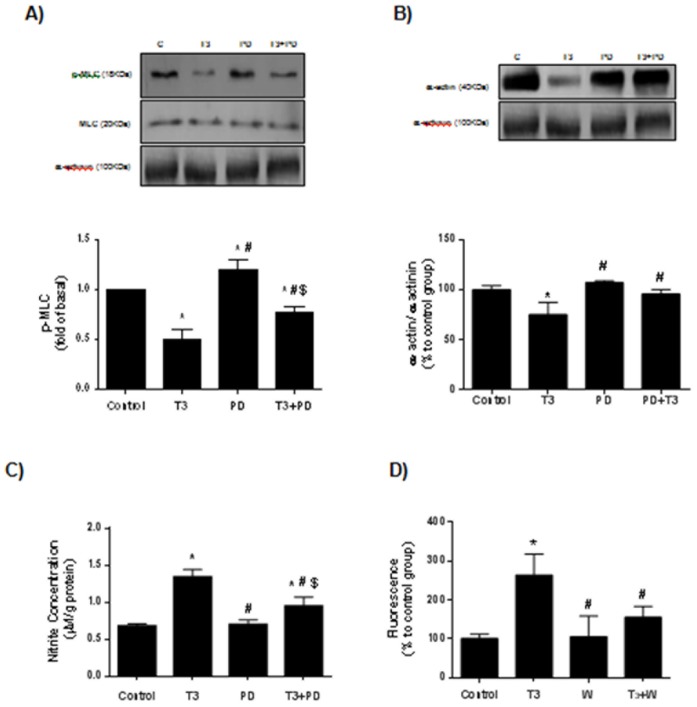
AT2R blocker reduces T3-induced decreased levels of contractile proteins and attenuates T3-induced NO production. Decreased levels of p-MLC (A) and α-actin (B) in VSMC treated with T3 (0.1 µM) for 24 h were attenuated in the VSMC pre-incubated with PD123319 (0.1 µM) for 30 min. Pre-incubation with PD123319 (0.1 µM) (C) and wortmannin (0.1 µM) (D) attenuates the T3-induced augmented NO generation in VSMC. In (C), NO_2_^−^ concentration was measured by chemiluminescence, while in (D) NO production was determined was determined using the NO-specific fluorescent dye 4,5-diaminofluorescein diacetate (DAF-2). Data are expressed as mean ± SD (n = 5 per group for A and B; n = 4 per group for C and D; *p<0.05 *vs.* control, #p<0.05 *vs.* T3, $p<0.05 vs. PD).

In order to investigate whether AT2R contributes to T3-induced vascular relaxation, cells were treated for 24 hours with T3 (0.1 µM) and/or pre-incubated with AT2R blocker. T3 induced a significant decrease in p-MLC and α-actin levels in cultured VSMC, which was partially reversed in cells pre-incubated with PD123319 for 30 min. These data support the previous results found in aortas from T3-treated rats and also show that the direct effect of T3 in diminishing the contractile apparatus proteins is mediated by AT2R (Figure 4).

### T3-induced NO Production Occurs via AT2R/Akt Pathway

**Figure 5:**
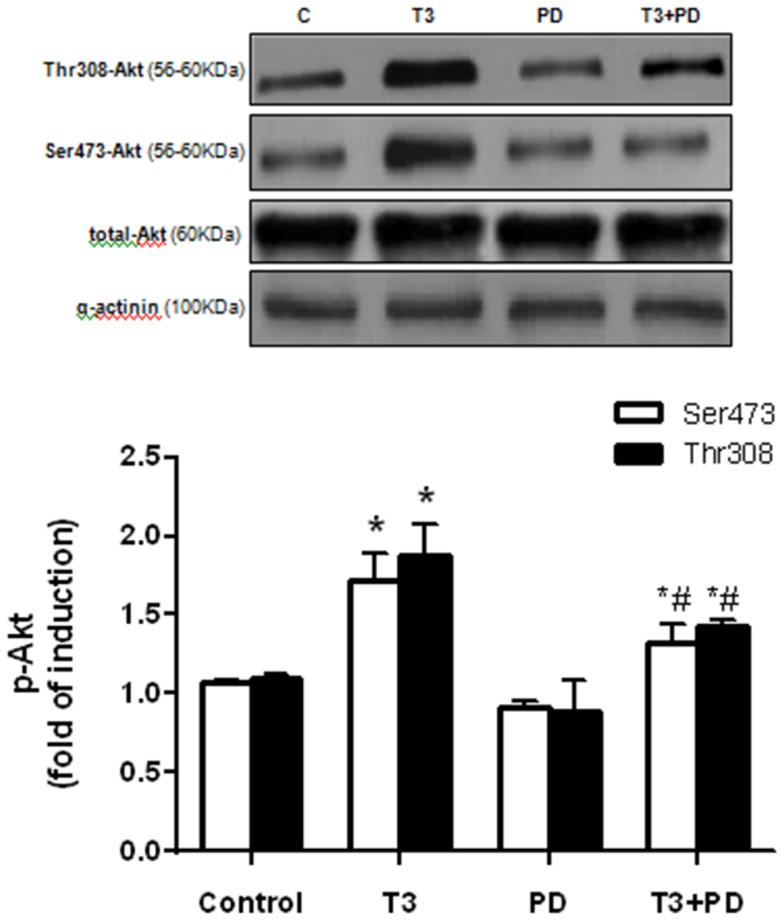
AT2R mediates T3-induced PI3K/Akt activation. Cultured VSMC stimulated with T3 (0.1 µM for 24 hours) display increased levels of p-Akt in both residues (thr308 and ser473), which is partially reversed by the specific blockade of AT2R with PD123319 (0.1 µM for 30 min prior to T3 treatment). Data are expressed as mean ± SD (n = 6 per group, *p<0.05 *vs.* control, #p<0.05 *vs.* T3).

**Figure 6:**
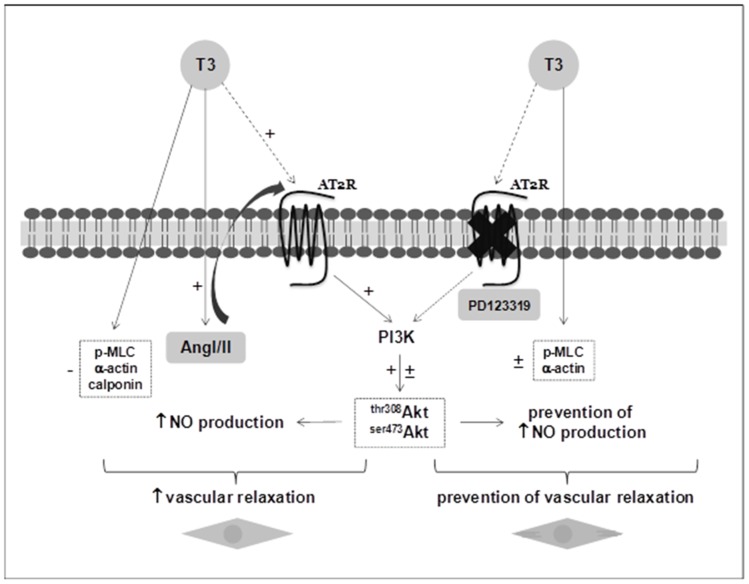
Schematic representation of the proposed model. T3 induces a sustained increase in AT2R and AngI/II levels, which in turn causes the activation of PI3K pathway with the consequent increment in Akt phosphorylation that finally leads to an augmentation in NO production. Besides, T3 also causes a diminution in contractile protein levels. Altogether, these effects promote an increase in vascular relaxation. On the other hand, the specific blockade of AT2R (with PD123319) is able to partially or completely abolish the effects of T3, culminating in the prevention of the increase of vascular relaxation and evidencing the role of AT2R/PI3K/Akt pathway in these processes.

We previously reported that T3 rapidly stimulates NO production in VSMC [5]. Using nitrite (NO^2−^) levels assay, we observed that the levels of NO^2−^ were augmented by 2 fold in supernatants of cultured VSMC stimulated with T3. This effect was diminished in cells pre-incubated with PD123319 (Figure 4C). Because the PI3K/Akt signaling pathway is downstream of AT2R [30] and is involved in the vascular NO production [24], we next examined the effects of wortmannin (W), a selective inhibitor of PI3K, in T3-induced NO production. The increased levels of NO in VSMC stimulated with T3 were partially reversed in the presence of wortmannin (Figure 4D). To confirm whether T3-induced PI3K/Akt activation occurs via AT2R, we measured activation of PI3K/Akt by phosphorylation in the presence of PD123319. We observed increased levels of phosphorylation Akt at Ser473 and Thr308 in VSMC stimulated with T3; however, in the presence of PD123319, this effect was reduced, showing that T3 activates PI3k/Akt signaling via AT2R (Figure 5). Together these results support the hypothesis that T3 stimulates AT2R in VSMC, which in turn activates PI3k/Akt signaling pathway that triggers NO production leading to vascular relaxation. Thus, we propose a new model to explain the decreased vascular contraction observed in the hyperthyroidism (Figure 6).

## Discussion

The novel finding of the current study was that AT2R present in VSMC plays a key role in the hyperthyroidism-induced decrease in contractile response accompanied by an increase in Akt/NO pathway signaling. This is based on the following results: a) Decreased contractile response to AngII in denuded aortas from hyperthyroid rats was attenuated by AT2R blocker; b) Decreased vascular contraction during hyperthyroidism was associated with increased levels of NO and activation of PI3K/Akt, both of which were attenuated through use of an AT2R blocker.

Unlike the well-known contribution of endothelium on vascular relaxation in hyperthyroidism [4], [31], there is a paucity of data on the role of VSMC in this field. Since thyroid hormone receptors (TLRs, TR-alpha and beta) have been identified in the coronary and aorta from human VSMC [32], the key question raised by these findings is whether TH has a direct effect in VSMC.

We recently showed that T3 rapidly leads to augment NO production in VSMC via PI3K/Akt pathway [5]; however the underlying mechanisms involved are still unknown. In addition, we and others investigators have previously demonstrated that cardiovascular actions promoted by TH occur with participation of local RAS [33], [34]. RAS is one of the most important regulators of vascular function and AT2R represents a crucial component responsible for vasodilation. Considering the possibility that AT2R might act as an upstream effector of NO production which in turn leads to decreased vascular contraction under hyperthyroidism condition, we hypothesized that AT2R in VSMC mediates the diminished vascular contraction in hyperthyroidism.

Herein, we used an experimental model of hyperthyroidism, in which the animals received daily an i.p. injection of T3 (20-fold the physiological dose) for 14 days. Initially, we confirmed the hyperthyroid status of the animals, who presented with a significant increase in heart rate. The tachycardia observed in hyperthyroidism occurs in part due to higher sensitivity of the cardiac tissue to catecholamines, since T3 induces an increase of β-adrenergic receptor expression [35]–[37]. Also, the positive chronotropic effect of TH is associated with altered expression of ion channels/transporters in the heart, such as Kv1.5, Kv4.2, hyperpolarization-activated cyclic nucleotide-gated channel 2 (HCN2), HCN4, Na-Ca^2+^ exchanger, and Na^+^-K^+^-ATPase [38]–[40]. As expected, systolic blood pressure (SBP) levels were increased in hyperthyroid animals, corroborating data of literature [41]. Consistent with the decrease in SVR observed in hyperthyroidism, we investigated the expression of contractile proteins in endothelium-denuded aorta from T3-treated animals. Aortas from hyperthyroid animals showed a significant decrease of phosphorylated myosin light chain (p-MLC) levels, a marker of vascular contractile status [42], [43]. Similarly, alpha-actin and calponin, essential proteins of contractile vascular machinery [44], [45], exhibited a significant reduction, indicating that hyperthyroidism alters the levels of these proteins and may contribute to changes in vascular tone leading to an increased vascular relaxation.

AT2R-mediated vasodilatation has been reported in both endothelium-dependent and independent manners [18], [19], [46], [47]. Based on AT2R upregulation observed in cardiomyocytes from the hearts of rats induced to hyperthyroidism [8] we evaluated the possible involvement of this receptor in VSMC with increased vascular relaxation observed in this pathology. We observed increased levels of AT2R in the aortas from hyperthyroid animals, corroborating with our initial hypothesis that AT2R might be involved in vascular relaxation found in hyperthyroidism. In addition, AngI/AngII protein expression levels were also significantly increased in aortas from hyperthyroid animals, indicating that T3 also increased the levels of the ligand AngII. The action of TH increasing local Ang II levels has already been described in other cell types [8], [9].

We also assessed the direct effect of T3 on cultured VSMC in order to investigate the possible molecular mechanisms involved in hormone action on relaxation response. According to the observations in endothelium-denuded aortas, AT2R was present under basal conditions in VSMC and its mRNA and protein expression levels were upregulated after T3 treatment with no changes on AT1R levels. Similarly, treatment of VSMC with T3 promoted a reduction in p-MLC levels in parallel with a decrease on alpha-actin levels, confirming the effect of T3 on reducing contractile proteins. This effect of TH on the contractile protein levels in VSMC has not been demonstrated previously in the literature. Taking into account recent evidence showing that AT2R stimulation could act on the contractile machinery modulating processes such as depolymerization of actin [48], we tested the hypothesis of a possible correlation between upregulation of AT2R and decreased contractile proteins found in VSMC stimulated with T3. Interestingly, VSMC treated previously with AT2R blocker (PD123319) partially reversed the effect of T3, showing for the first time an involvement of this receptor in the action of T3 in the reduction of contractile proteins, possibly to increase the vascular relaxation found in the hyperthyroidism.

Based on results obtained *in vitro* we performed functional studies using vascular reactivity experiments in order to evaluate the role of AT2R on relaxation induced by T3. Endothelium-denuded aorta rings from hyperthyroid animals presented a lower response to Ang II, compared to control. This effect was partially reversed when the rings were pre-incubated with PD123319, indicating that the lower contractile response occurs at least in part to the involvement of AT2R. In fact, previous studies demonstrated that AT2R located in smooth muscle may directly mediate vasodilatation effects, but also may minimize the contraction promoted by Ang II [49]. Besides the morphological arrangement and all the vessel contractile components, aorta is responsive to numerous vascular modulators including TH, which makes it a good model to study vascular contractile or relaxation phenomena. In addition, this vessel serves as an important tool to obtain primary cultures of VSMC [50].

One of the mechanisms by which AT2R promotes vasodilatation is through nitric oxide production (NO) [18], [29], which seems to occur after stimulation of bradykinin by AT2R [51], [52]. Thus, an assay to indirectly evaluate the NO levels through the nitrite concentrations determination [25] was performed in cultured VSMC submitted to T3 treatment in the presence or absence of AT2R blocker. T3 promoted an increase in nitrite concentration which was partially reversed after pretreatment with the AT2 blocker. Increased vascular NO production has been reported in rats in hyperthyroid states or after treatment with T3 for 3–4 months [4]. In addition, both nNOS and eNOS have been identified as targets for the actions of TH [53].

Based on studies showing activation of Akt signaling by TH in other cell types [9] and a non-genomic effect of TH in increasing NO levels [5], we also found impairment in the NO production induced by T3 when an Akt inhibitor (wortmannin) was used. It has been recently demonstrated that the Akt pathway, classically activated by AT1R [54], may also be stimulated upon AT2R activation [30]. In the present study we showed that the AT2R blockade partially prevented the increase on Akt phosphorylation levels promoted by T3. Although we didn’t have analyzed which NOS is involved in this process, it is possible that T3-induced NO production by VSMCs may occur with expressive participation of inducible and neuronal NOS as showed previously [5].

In summary, this study demonstrated by *in vivo* and *in vitro* assays a novel mechanism associated with the decreased vascular contraction promoted by thyroid hormone demonstrating a role of AT2R/Akt/NO pathway. We showed in the present study an interaction between TH and vascular RAS mediating vascular function.
